# Research on Super-Resolution Reconstruction of Coarse Aggregate Particle Images for Earth–Rock Dam Construction Based on Real-ESRGAN

**DOI:** 10.3390/s25134084

**Published:** 2025-06-30

**Authors:** Shuangping Li, Lin Gao, Bin Zhang, Zuqiang Liu, Xin Zhang, Linjie Guan, Junxing Zheng

**Affiliations:** 1 School of Geodesy and Geomatics, Wuhan University, Wuhan 430079, China; 2 Changjiang Spacial Information Technology Engineering Co., Ltd., Wuhan 430010, China; 3 Hubei Provincial Research Center for Hydraulic Information Perception and Big Data Engineering Technology, Wuhan 430010, China; 4 National Dam Safety Engineering Technology Research Center, Wuhan 430010, China; 5 School of Civil and Hydraulic Engineering, Huazhong University of Science and Technology, Wuhan 430074, China

**Keywords:** Real-ESRGAN, super-resolution reconstruction, coarse granular materials, particle image enhancement, wavelet transform, image degradation modeling, earth/rock dam construction

## Abstract

This paper investigates the super-resolution reconstruction technology of coarse granular particle images for embankment construction in earth/rock dams based on Real-ESRGAN, aiming to improve the quality of low-resolution particle images and enhance the accuracy of particle shape analysis. The paper begins with a review of traditional image super-resolution methods, introducing Generative Adversarial Networks (GAN) and Real-ESRGAN, which effectively enhance image detail recovery through perceptual loss and adversarial training. To improve the generalization ability of the super-resolution model, the study expands the morphological database of earth/rock dam particles by employing a multi-modal data augmentation strategy, covering a variety of particle shapes. The paper utilizes a dual-stage degradation model to simulate the image degradation process in real-world environments, providing a diverse set of degraded images for training the super-resolution reconstruction model. Through wavelet transform methods, the paper analyzes the edge and texture features of particle images, further improving the precision of particle shape feature extraction. Experimental results show that Real-ESRGAN outperforms other traditional super-resolution algorithms in terms of edge clarity, detail recovery, and the preservation of morphological features of particle images, particularly under low-resolution conditions, with significant improvement in image reconstruction. In conclusion, Real-ESRGAN demonstrates excellent performance in the super-resolution reconstruction of coarse granular particle images for embankment construction in earth/rock dams. It can effectively restore the details and morphological features of particle images, providing more accurate technical support for particle shape analysis in civil engineering.

## 1. Introduction

Earth/rock mixtures, due to their broad applicability, are widely used in highway subgrade and water conservancy projects. The engineering properties of these mixtures are influenced by particle shape and roundness, which directly impact the compaction, stability, and mechanical properties of the mixture. Therefore, strict control of particle shape and roundness is essential to ensure material performance and project quality during construction.

Currently, the automatic classification and statistical analysis of the appearance characteristics of earth/rock mixture particles mainly rely on image recognition technology. Particle images are captured using imaging devices, and features are extracted to determine the shape and roundness of the particles. Image resolution is a key indicator of image quality; the higher the resolution, the clearer the image details and texture [1], which helps to more accurately identify particle characteristics.

However, due to factors such as imaging devices and external environments, images are prone to distortion during acquisition, transmission, and storage, which often fail to meet practical application requirements [2]. To address this, image super-resolution (SR) reconstruction technology has emerged. This technology enhances the resolution and texture quality of low-resolution (LR) images through software algorithms, generating high-resolution (HR) images without the need to modify existing imaging devices or being affected by transmission processes. It is cost-effective and suitable for real-world production and application scenarios.

### 1.1. Image Super-Resolution Reconstruction Algorithms for Ideal Degradation Models

Traditional Convolutional Neural Networks (CNN) have demonstrated powerful feature extraction capabilities in image super-resolution [3]. Representative methods include SRCNN [4], which uses bicubic interpolation followed by convolutional mapping, and ESPCN [5], which adopts sub-pixel convolution for more efficient upsampling. Later advancements such as VDSR [6], EDSR [7], and RCAN [8] introduced residual learning, network simplification, and attention mechanisms to further enhance performance.

While these methods generally optimize for Peak Signal-to-Noise Ratio (PSNR), they tend to produce overly smooth images, resulting in the loss of high-frequency texture and edge details critical to morphological recognition.

### 1.2. Image Super-Resolution Reconstruction Algorithms for Real Degradation Models

To overcome high-frequency detail loss in ideal degradation models, Generative Adversarial Network (GAN)-based methods were introduced for more realistic texture generation. SRGAN [9] was the first to apply GANs [10] in super-resolution, combining adversarial and perceptual losses [11] to improve texture realism and structural fidelity. ESRGAN [12] built upon this by incorporating dense residual blocks [13] and refining both perceptual and adversarial losses. Later works such as RankSRGAN [14] and SPSR [15] introduced ranking loss and structural priors, respectively, to better align with human perception and structural consistency.

Despite these improvements, GAN-based models still suffer from instability and detail degradation under high upscaling factors. To address complex real-world degradations, BSRGAN, proposed by Zhang et al. [16], introduces a flexible single-stage degradation model with randomized blur, downsampling, and noise. FeMaSR, developed by Chen et al. [17], improves realism through feature-domain alignment of distorted LR and reference HR features.

Recent developments have further extended this line of research. For instance, SeeSR [18] utilizes semantic priors to enhance region-specific consistency, while SeD [19] introduces degradation-aware discriminators to better recover fine-grained details under real-world conditions. More recently, diffusion-based models [20] have gained popularity for their ability to model complex distributions in generative tasks. Their theoretical foundations and broad application landscape are thoroughly reviewed in [21], providing useful insights for their adoption in super-resolution.

Despite their advantages, many of these advanced models require extensive computational resources and are tailored primarily for natural image benchmarks. In contrast, this study adopts Real-ESRGAN [22], which achieves a favorable trade-off between perceptual quality and inference efficiency. By employing a dual-stage degradation pipeline and a U-Net-based discriminator, Real-ESRGAN improves robustness while remaining practical for field-oriented applications such as soil particle morphology reconstruction, where textures are complex but semantic structures are limited.

In summary, both CNN and GAN methods have achieved significant results in image super-resolution reconstruction. Despite the notable progress made by existing algorithms such as SRCNN and ESRGAN in improving perceptual quality and detail recovery, issues like detail loss and artifacts remain, especially in high-magnification super-resolution tasks. Moreover, some methods have made breakthroughs in modeling complex degradation processes in real-world scenarios, enabling them to simulate various degradation conditions and enhance the quality of reconstructed images. Future research is expected to focus on enhancing model stability, recovering fine-grained textures under severe degradation, and improving generalization performance across varying degradation types and domains.

## 2. Dataset Construction

### 2.1. Particle Image Collection and Classification

**Figure 1:**
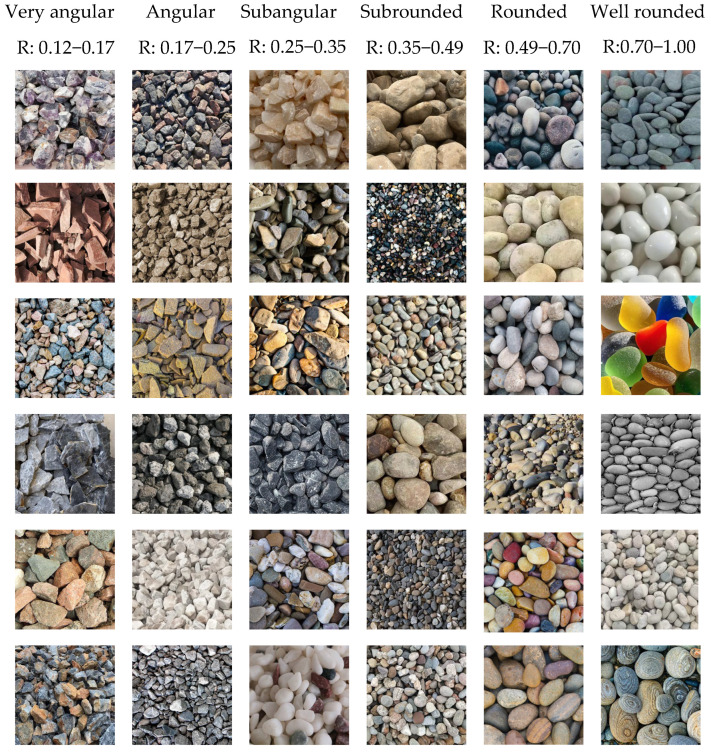
Partial image dataset.

In this study, a standardized process was used to construct a morphological database of earth/rock dam particles. Representative engineering samples were collected from six geographic and climatic regions in China. After sieving (particle size 5–200 mm) and surface cleaning, the images were captured using a Nikon D850 camera in a vertical overhead position, with all images having a resolution of 4090 × 4090 pixels. Particle roundness was calculated using ImageJ (v1.54f, National Institutes of Health, Bethesda, MD, USA), and combined with visual evaluation by three geological engineers, 550 valid samples were classified into six categories: very angular (0.12–0.17), angular (0.17–0.25), subangular (0.25–0.35), subrounded (0.35–0.49), rounded (0.49–0.70), and well rounded (0.70–1.00). Typical sample morphological features and roundness distribution are shown in Figure 1. All particle images were captured as single frames under static laboratory conditions, with no temporal sequences available for multi-frame processing.

### 2.2. Data Augmentation and Quality Thresholds

**Figure 2:**
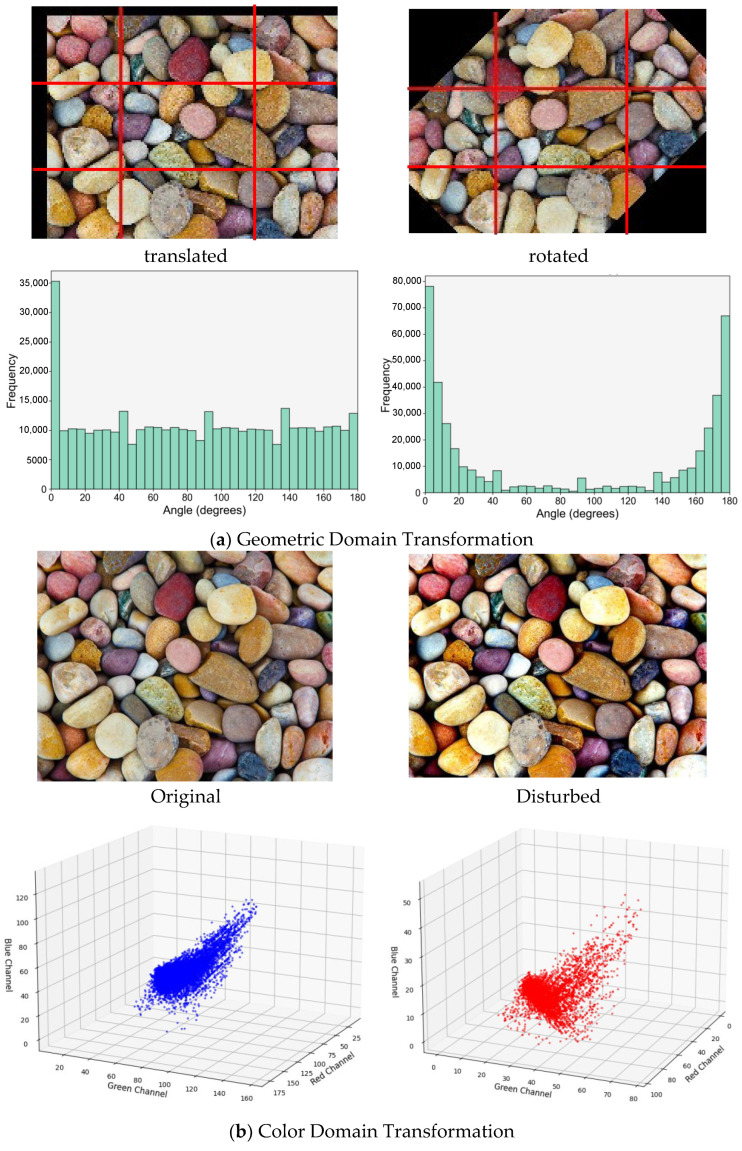
Image Augmentation Methods.

To enhance the generalization performance and robustness, a comprehensive data augmentation pipeline was employed. In the geometric domain, operations such as random cropping (size 2045 × 2045 pixels), horizontal, vertical, and mirror flips (each with a probability of 0.5), flips, ±30° rotations, and ±15% translations were applied. In the color domain, brightness adjustment (±50%), contrast stretching (γ ∈ [0.5, 1.5]), and RGB perturbation (saturation ±0.25) were implemented. The augmentation process increased texture complexity by 2.8 times and expanded the dataset to 1540 images (Figure 2). This increase was quantified using a gray-level co-occurrence matrix-based complexity metric, which demonstrated stronger sensitivity to local spatial variations than entropy or variance-based alternatives. As shown in Figure 2a, the geometric transformation increased the dispersion of edge direction distribution by 37.5%, indicating enhanced structural diversity. In Figure 2b, color perturbation resulted in a 21.3% decrease in RGB channel correlation, expanding the color space coverage of the dataset.

**Figure 3:**
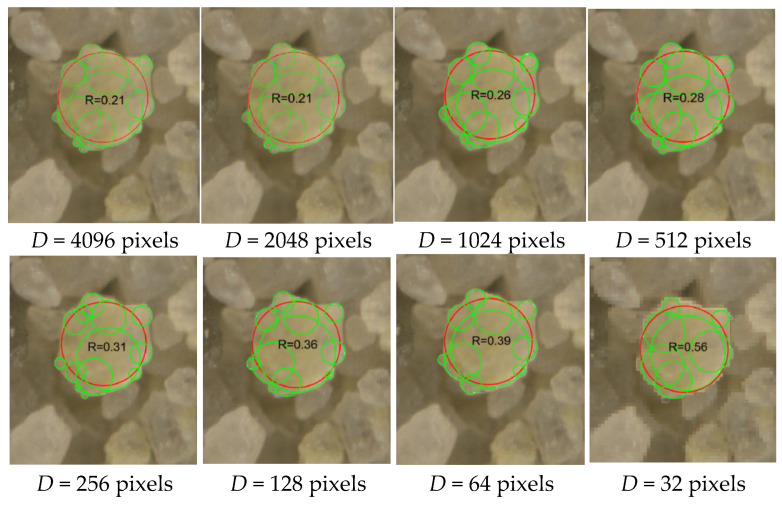
Image Quality on the Accuracy of Particle Shape Analysis. Fitted corner circles (green) and maximum inscribed circles (red).

To ensure sufficient resolution for shape analysis, especially roundness, minimum image quality thresholds were investigated. Figure 3 illustrates that low-resolution images cause large roundness deviations. This effect is especially pronounced in dense particle assemblies where particles are in contact, making it difficult to distinguish boundaries. Using over 300 manually measured particles per image, a critical particle size threshold of ~130 pixels was established to ensure consistent morphological metrics.

### 2.3. Dual-Stage Image Degradation

To simulate realistic degradation encountered during image acquisition and transmission, a dual-stage degradation model was implemented and is mathematically defined as:

(1) $$I_{\mathrm{LR}}^{\left(2\right)}=D_{2}(D_{1}(I_{\mathrm{HR}}))$$

where *I*_HR_ is the high-resolution input image, and *D*_1_, *D*_2_ denote sequential degradation operations. First Stage *D*_1_: Generate the initial degraded image $I_{\mathrm{LR}}^{\left(1\right)}$; Second Stage *D*_2_: Further apply blurring, noise, downsampling, and compression artifacts on $I_{\mathrm{LR}}^{\left(1\right)}$, where D(·) represents the degradation model. Each stage includes blurring, downsampling, noise addition, and JPEG compression, with parameter variations to represent diverse real-world scenarios.

**Figure 4:**
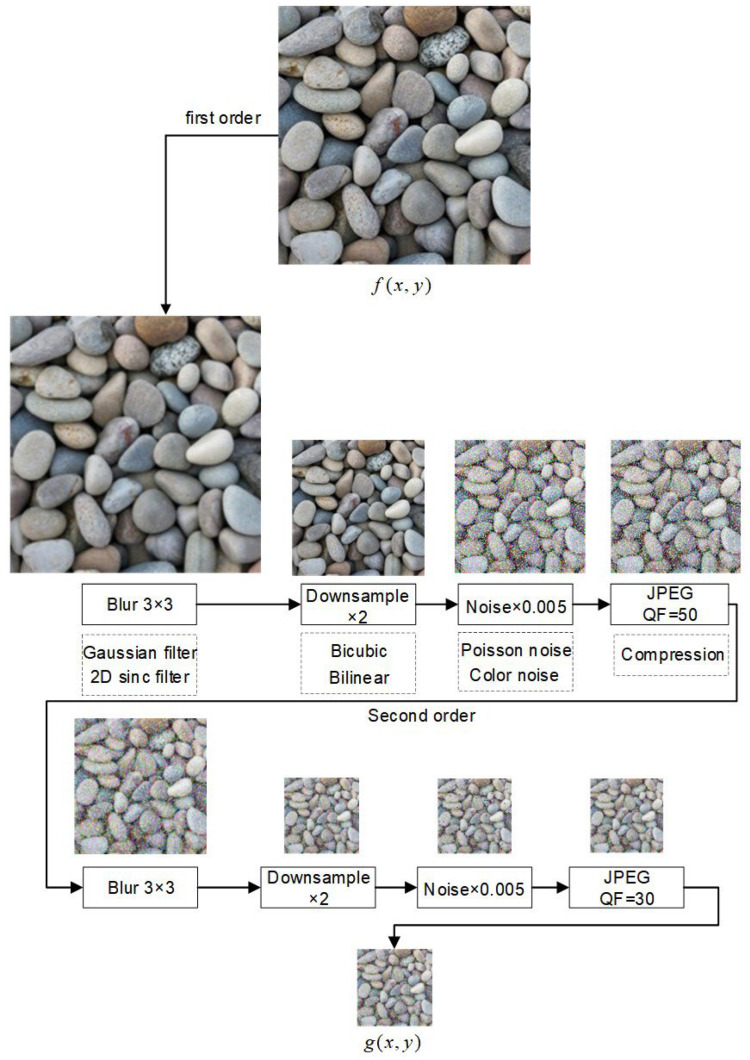
Dual-Stage Degradation Flow Diagram.

The detailed degradation pipeline is illustrated in Figure 4. This order of operations reflects the typical image degradation process in real-world scenarios: optical or motion blur usually occurs first during acquisition, followed by resolution reduction due to sensor limitations, noise introduced by sensors or environment, and finally, compression artifacts during storage or transmission. This sequential design is also adopted in prior works such as BSRGAN [16], enhancing realism and consistency in low-resolution image simulation. In the first stage, a Gaussian blur with a 3 × 3 kernel is applied to mimic optical or motion blur, followed by 2× downsampling using bicubic or bilinear interpolation. Gaussian or Poisson noise with a standard deviation of σ = 0.005 is added, and JPEG compression with a quality factor (QF) of 50 is used to emulate acquisition-level compression. The second stage applies a similar process with more severe settings: a larger blur kernel, increased noise variance, and JPEG compression with QF = 30 to simulate transmission-related degradation under bandwidth or storage constraints.

The Gaussian blur operation is governed by the kernel k(i,j):

(2) $$k(i,j)=\frac{1}{N}\exp\left(-\frac{1}{2}C^{T}\Sigma^{-1}C\right),\;C={\left[i,j\right]}^{T}$$

where Σ is the covariance matrix of the Gaussian kernel, which controls the blur range and direction; C is the center point coordinates of the kernel; N is the normalization factor, ensuring that the sum of the Gaussian kernel equals 1.

The blurred image *I_blur_* is obtained by convolution:

(3) $$I_{\mathrm{blur}}=I_{\mathrm{HR}}*k$$

where ∗ represents the convolution operation.

Gaussian noise is added to the image to simulate sensor noise or environmental interference in real-world scenarios:

(4) $$I_{\mathrm{noisy}}=I_{\mathrm{down}}+n,\;n\sim N(0,\sigma^{2})$$

where $\sigma^{2}$ controls the intensity of the noise.

JPEG quality factors are deliberately set at QF = 50 and QF = 30 in the two stages to emulate realistic degradation accumulation. The general quality factor range remains q ∈ [10, 100], and Figure 4 visualizes the full degradation pathway.

It is worth noting that JPEG compression is itself a compound transformation process consisting of block-wise discrete cosine transform, quantization, and entropy coding. These steps introduce characteristic artifacts such as blocking and ringing, making JPEG particularly suitable for simulating realistic transmission-related degradation.

### 2.4. Minimum Image Quality for Reliable Optical Characterizations of Soil Particle Shapes

Image-based techniques have revolutionized the analysis of soil particle morphology by significantly improving efficiency and objectivity compared to traditional manual techniques. However, geotechnical practitioners often overlook the sensitivity of these methods to input quality. When inconsistencies arise, the blame is typically attributed to particle heterogeneity rather than to the limitations of the image-based computational techniques. However, image quality, particularly resolution, plays a critical role in the reliability of shape descriptors.

**Figure 5:**
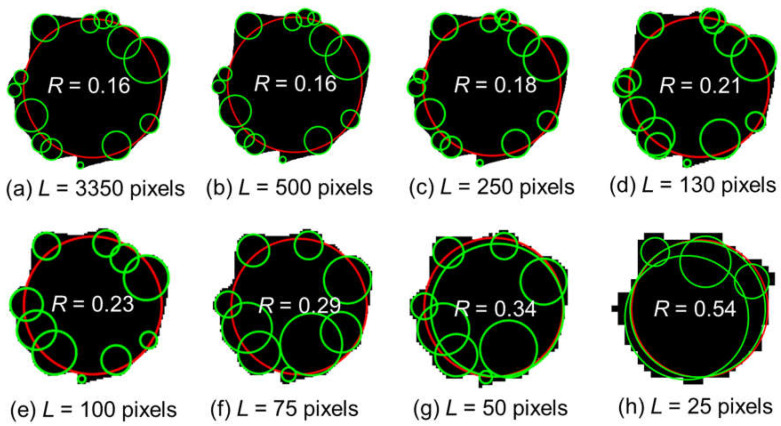
Impact of image quality on the accuracy of particle shape analysis.

As demonstrated in Figure 5, particles captured at different resolutions (from 3350 to 25 pixels) exhibit increasing levels of aliasing and edge distortion as resolution decreases. Low-resolution images obscure sharp corners and intricate boundary features, leading to significant overestimation of descriptors such as roundness. These deviations are not minor. For example, a roundness deviation of merely 0.1 has been shown to induce a 1.7° shift in the predicted critical state friction angle [23] and a 2.4° alteration in the peak friction angle [24], highlighting the sensitivity of strength parameters to shape characterization and the resulting implications for geotechnical design reliability.

Sun et al. [25] provided a quantitative investigation into how various shape descriptors respond to reductions in image quality, establishing minimum resolution requirements for reliable analysis. They used particle length *D* (in pixels) as the reference indicator and classified descriptors by their sensitivity to degradation. Coarse descriptors such as aspect ratio (AR) and shape complexity (SC), which rely primarily on basic dimensions like length and width, proved resilient to resolution loss. Medium-coarse descriptors, including surface diameter (SD) and area sphericity (SA), which depend on areal measurements, also retained acceptable accuracy at moderate resolutions.

In contrast, fine and very fine descriptors that rely on detailed perimeter definitions, such as perimeter sphericity (SP), convexity, and especially roundness (R), demonstrated substantial degradation in accuracy as resolution declined. These metrics are particularly vulnerable to edge blurring and aliasing and, thus, require high-resolution images to preserve the fidelity of boundary features. Table 1 summarizes the minimum particle length *D*_min_ needed to ensure accurate characterization across different shape descriptor categories.

These findings emphasize that not all shape descriptors demand the same image quality. Analysts should select image resolutions based on the type of shape information they intend to extract. While coarse descriptors may tolerate moderate resolution loss, accurate extraction of roundness and similar high-sensitivity metrics necessitates maintaining a minimum image resolution, thereby ensuring the reliability and interpretability of computational shape assessments in geotechnical applications.

### 2.5. The Necessity of Minimum Image Quality for Analyzing Particle Shapes in Assemblies

Previous studies on image quality standards for soil particle shape analysis have predominantly focused on binary images of isolated particles. For instance, Sun et al. [25] established a hierarchy of resolution thresholds based on the particle length *D* to ensure reliable computation of various shape descriptors. These thresholds were grounded in systems where particles are clearly separated prior to imaging, such as the University of Illinois Aggregate Image Analyzer [26,27], the Aggregate Imaging System [28,29,30], and the Translucent Segregation Table [31].

In contrast, several modern imaging systems, such as the Sedimaging system and the Vision Cone Penetrometer developed by Raschke and Hryciw [32], Ghalib et al. [33], Hryciw and Ohm [34], and Zheng and Hryciw [35], capture particles in dense three-dimensional assemblies. In these systems, particles are not physically separated, resulting in occlusion and overlapping projections that complicate individual particle recognition. This challenge is especially pronounced when imaging fine sand particles, which are difficult to isolate even under controlled laboratory conditions.

**Figure 6:**
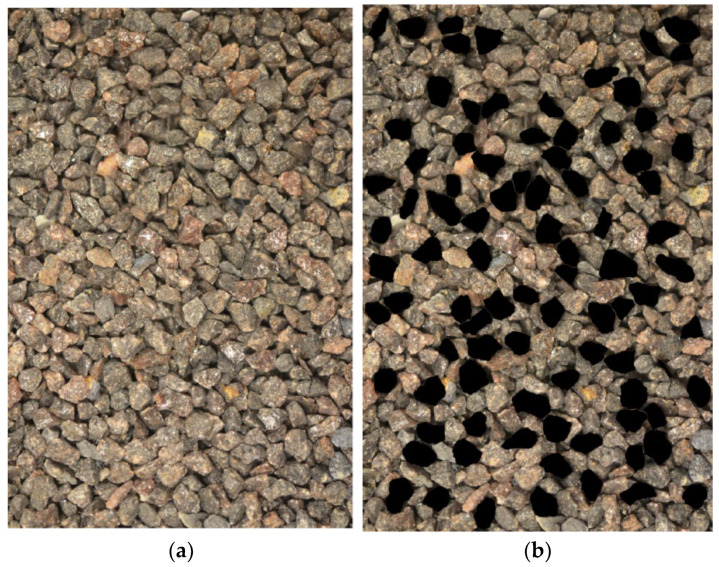

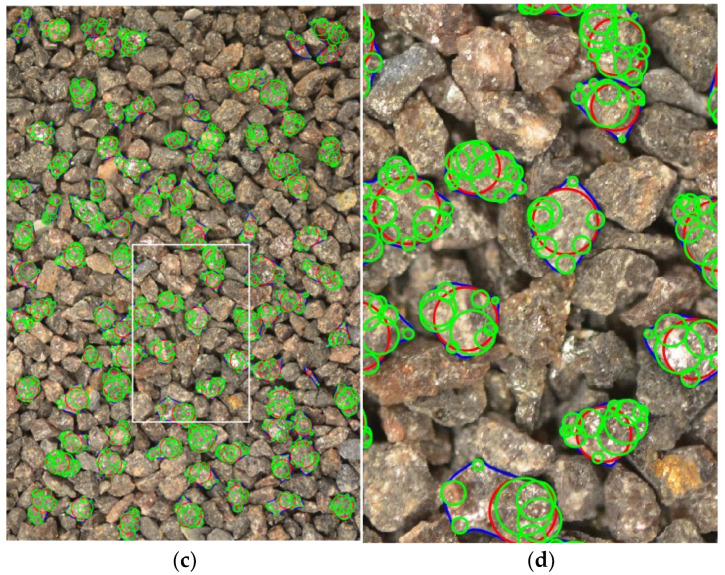
Circle fitting results for BFAO sand: (**a**) image of the three-dimensional assembly of BFAO; (**b**) delineated particles using Photoshop polygonal lasso tool; (**c**,**d**) fitted corner circles (green) and maximum inscribed circles (red).

To overcome this limitation, a semi-automated image processing strategy is applied. For instance, Figure 6a presents a typical image of Brown Fused Alumina Oxide (BFAO) sand, where particles display complex surface textures and appear in a compact 3D arrangement. In such cases, automated segmentation often fails to identify fully visible particles. Therefore, an operator uses image editing software (e.g., Photoshop) to manually trace particle boundaries using the polygonal lasso tool, selecting only those particles that exhibit clear, unobstructed projections. As shown in Figure 6b, the selected regions are filled with unique colors to generate particle masks, which are then input into a computational geometry program. This program extracts the particle contours, fits corner circles and maximum inscribed circles, and computes shape descriptors such as roundness, as demonstrated in Figure 6c,d.

The quality of images significantly impacts the accuracy of particle shape analysis in sand assemblies, as illustrated in Figure 3. Variations in roundness calculations become evident when images are captured at different resolutions, emphasizing how image quality influences the reliability of morphological analysis. This highlights the potential for inaccurate results from low-resolution images, underscoring the importance of ensuring high image quality and resolution, especially when analyzing complex particle arrangements where particles are in contact. Consistent and reliable shape descriptors depend on this.

While the image quality standards outlined by Sun et al. [25] remain valuable for analyzing particle shapes in sand clusters, a key challenge arises when determining the particle length *D*. In binary images where particles are isolated, size calculations are straightforward. However, in assembly images, where particles often touch or overlap, precise measurements become more challenging. To address this issue, the study introduces wavelet analysis, a method capable of isolating individual particles in densely packed assemblies, enabling more accurate size determination for shape analysis.

## 3. Wavelet-Based Image Quality Assessment and Resolution Metrics

### 3.1. Basic Principle of Wavelet Analysis

The Haar wavelet transform has gained widespread acceptance in various applications due to its straightforward implementation and minimal computational complexity [36,37,38,39]. It is particularly effective in identifying sharp intensity variations, which are commonly associated with particle edges or boundaries in digital images. For soil particle analysis, the accurate detection of these boundaries is essential for determining the dimensional characteristics of individual particles. This method thus provides a solid foundation for particle size analysis in soil mechanics studies.

**Figure 7:**
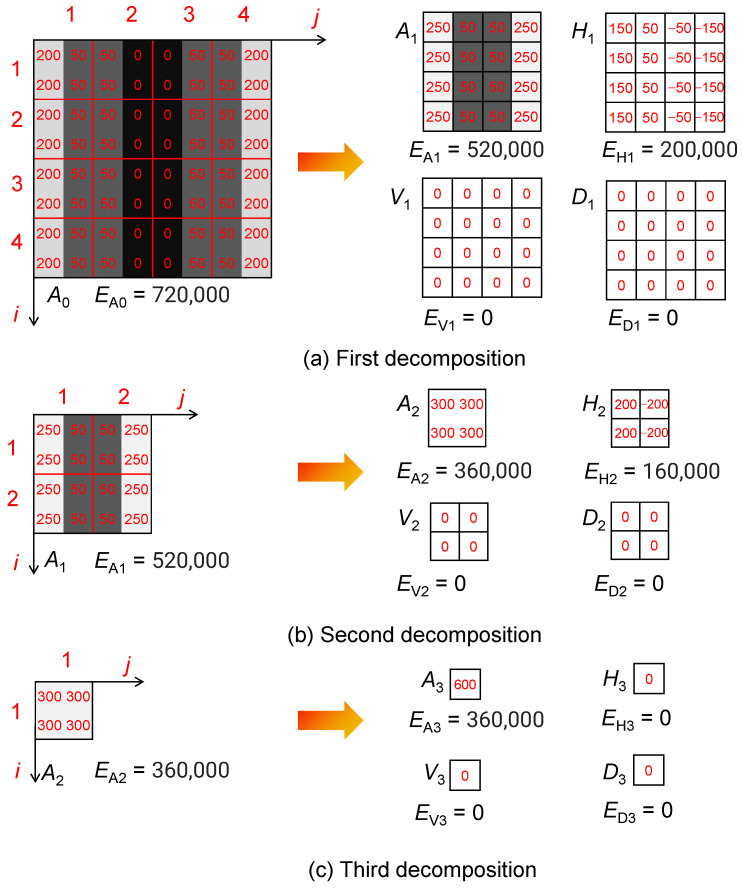
Illustration of the Haar Wavelet Transform Principle.

The Haar wavelet transform operates by decomposition the images into smaller sub-regions, with each sub-region capturing local features. In the case of a 2 × 2 pixels block, four coefficients representing the average values and intensity gradients are computed to describe the local image features [40,41]. In Figure 7, an 8 × 8 monochrome image *A*_0_ is shown, where variations in pixel intensity are plotted across the matrix. The brighter regions indicate higher intensity values, and in this specific setup, intensity gradients appear predominantly in the horizontal direction, showing uniformity along the vertical axis.

The decomposition process starts by segmenting the original image into 16 non-overlapping 2 × 2 pixels blocks. Each block is processed to generate localized wavelet coefficients. This hierarchical breakdown allows for efficient detection of directional intensity changes, which is crucial for edge localization and texture identification.

In Figure 7, the Haar-based decomposition results are illustrated. The average luminance of each block is computed as component *A*_1_, which serves as an indicator of the image’s general brightness:

(5) $$A_{1}(i,j)=\frac{A_{0}(i,j)+A_{0}(i+1,j)+A_{0}(i,j+1)+A_{0}(i+1,j+1)}{2}$$

Alongside this, three additional components are calculated.

A lateral gradient metric (*H*_1_) isolates horizontal intensity variations by comparing left and right sub-block segments:

(6) $$H_{1}(i,j)=\frac{A_{0}(i,j)-A_{0}(i+1,j)}{2}$$

An axial differential term (*V*_1_) quantifies vertical intensity discrepancies between upper and lower partitions:

(7) $$V_{1}(i,j)=\frac{A_{0}(i,j)-A_{0}(i,j+1)}{2}$$

and a diagonal contrast parameter (*D*_1_) captures oblique intensity variations across opposing corners:

(8) $$D_{1}(i,j)=\frac{A_{0}(i,j)-A_{0}(i+1,j+1)}{2}$$

The discrete wavelet transform decomposes the original image into four coefficient matrices that capture different spatial/frequency components: an approximation matrix (*A*_1_) and three detail matrices (*H*_1_, *V*_1_, and *D*_1_). As shown in Figure 7a, *A*_1_ represents the low-frequency content and serves as a downsampled version of the original image *A*_0_, reducing the resolution by half. The detail matrices *H*_1_ and *V*_1_ capture horizontal and vertical intensity variations, respectively. In the specific example presented, the vertical detail matrix (*V*_1_) contains zero values, indicating no vertical gradient due to the design of the test image. *D*_1_ encodes diagonal changes and completes the decomposition.

The Haar basis was selected in this study due to its step-like structure, which enhances edge localization and yields interpretable directional features. This characteristic makes it particularly suitable for coarse, angular particle morphologies, where higher-order spline-based wavelets (e.g., Daubechies) may lead to over-smoothing and diminished boundary sharpness.

The energy across these components is quantified by the squared Frobenius norm of each coefficient matrix. This yields energy parameters *E*_A1_, *E*_H1_, *E*_V1_, and *E*_D1_, which capture the energy associated with horizontal, vertical, and diagonal gradients. The principle of energy conservation is upheld, ensuring that the total energy of the original image (*A*_0_) is equivalent to the sum of the energies of its components.

(9) $$E_{A0}=E_{A1}+E_{H1}+E_{V1}+E_{D1}$$

The decomposition process proceeds by analyzing the approximation subband *A*_1_, as seen in Figure 7b. The iterative refinement continues, producing additional sets of transform coefficients *A*_2_, *H*_2_, *V*_2_, and *D*_2_, which further isolate intensity gradients along orthogonal spatial axes.

At each level of Haar decomposition, energy metrics such as *E*_H_, *E*_V_, and *E*_D_ quantify intensity variations along specific orientations. These values reflect brightness fluctuations that become increasingly structured over larger receptive fields. For example, at level 2, *E*_H2_ and *E*_V2_ measure gradients over 2 × 2-pixel regions, while at level 3, the receptive field expands to 4 × 4 pixels, enhancing the capture of broader structural patterns. This multiscale representation facilitates the detection of particle edges and texture boundaries.

**Figure 8:**
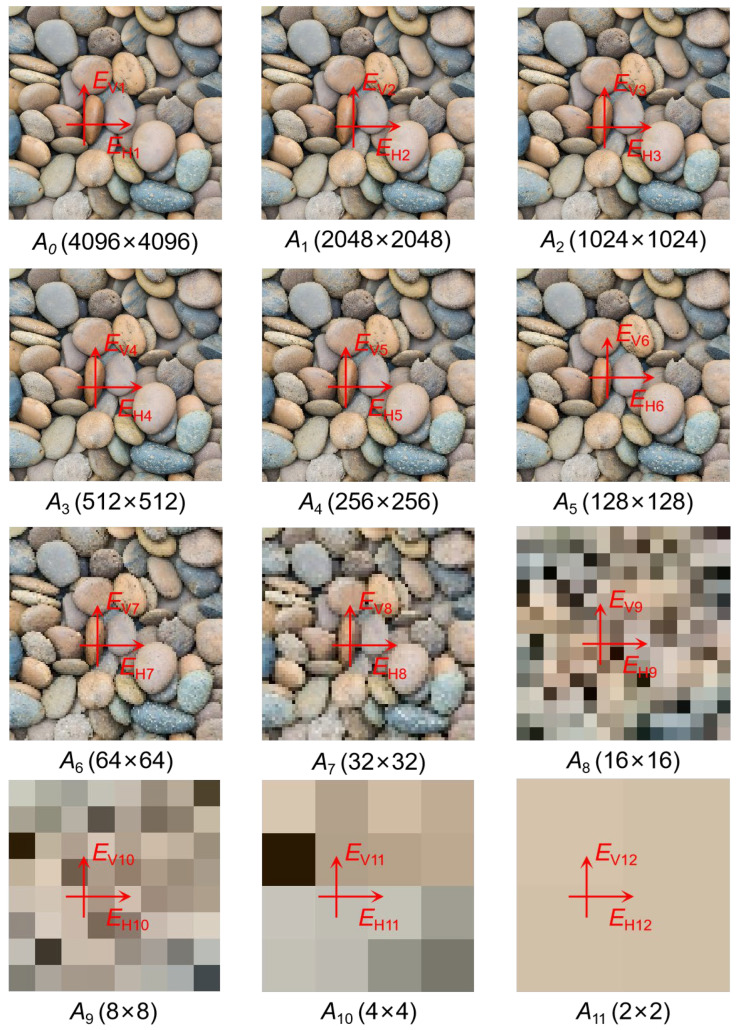
Representative Soil Particle Images and Results of Haar Wavelet Decomposition Analysis.

The decomposition proceeds by iteratively analyzing the approximation subband, isolating directional changes and gradually filtering out fine-scale details. For a source image of 4096 × 4096 pixels, this process supports up to 12 decomposition levels, successively halving resolution and suppressing high-frequency content associated with texture. Figure 8 shows the original image *A*_0_ and representative decomposition results up to *A*_11_, where each stage corresponds to a refined spatial scale.

**Figure 9:**
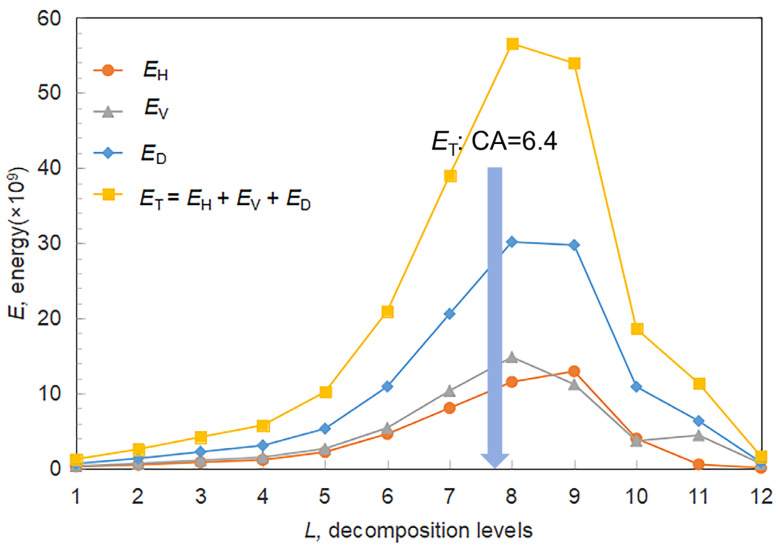
Variation of wavelet subband energy with decomposition level.

Figure 9 presents the variations of energy metrics (*E*_H_, *E*_V_, and *E*_D_) as a function of decomposition level *L*. Energy levels initially increase, reaching a peak around *L* = 8, then decline as particle boundaries become increasingly blurred. This peak corresponds to the scale at which boundary features are most distinct and is closely related to the dominant particle size in the image dataset.

The peak energy at level 8 corresponds to a composite coefficient *CA* = 6.4, which serves as a reference for optimal feature extraction. During early decomposition levels (*L* < 8), the removal of fine textures enhances boundary contrast, resulting in energy amplification. Beyond this point, continued decomposition suppresses edge-related detail, leading to reduced energy values and lower gradient magnitudes.

This relationship between energy and level follows a parabolic trend. The vertex of this curve indicates the scale at which particle boundaries contribute most significantly to image structure. Analytical modeling shows that coarser particle distributions shift this vertex to higher *L* values, aligning with expected spatial characteristics. However, since discrete wavelet transforms operate on integer levels, this introduces quantization effects that limit fine-grained granularity estimation.

To address this, a weighted averaging method approximates the decomposition level as a continuous value. Specifically, the composite coefficient *CA* proposed by Shin and Hryciw [42] enables sub-level precision through interpolation. The decomposition level is denoted by *i*, and an image with dimensions 2*^L^* × 2*^L^* pixels can be decomposed up to *L* levels. The total energy at level *i*, denoted as *E*_Ti_, is calculated by aggregating the squared Frobenius norms of the detail components:

(10) $$E_{Ti}=E_{Ei}+E_{Vi}+E_{Di}$$

The optimal level *CA* is derived as the energy-weighted average of all decomposition levels:

(11) $$CA=\frac{\sum_{i=1}^{L}i\times E_{Ti}}{\sum_{i=1}^{L}E_{Ti}}$$

This approach preserves the image’s energy structure and supports more accurate estimation of dominant spatial features, especially for particle morphology analysis.

### 3.2. Determine the Mean Particle Length by Wavelet Analysis

**Figure 10:**
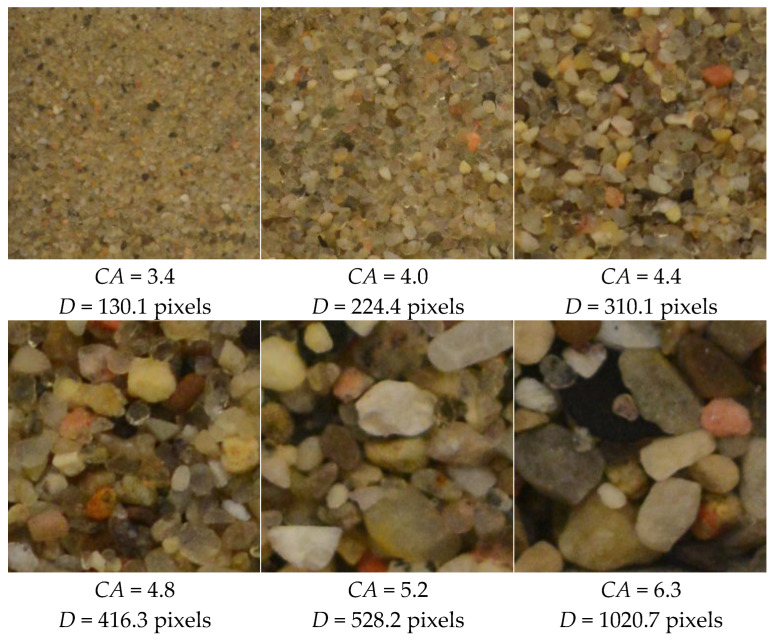
Relationship between wavelet coefficient *CA* and particle size *D.*

To validate the utility of wavelet coefficients in characterizing particle size, a standard sand sample (2NS) was sieved into multiple size groups and imaged at a fixed resolution of 4096 × 4096 pixels. As shown in Figure 10, the Haar wavelet transform was applied to each image, and the corresponding composite coefficient *CA* was calculated using Equations (12) and (13). Manual measurements of average particle length *D* were manually measured by selecting at least 300 fully visible particles per image.

Results show a clear positive correlation between *CA* and *D*, indicating that larger particle sizes are associated with higher wavelet coefficient values. This trend suggests that *CA* can serve as a reliable proxy for average particle length, enabling rapid and objective assessment of image quality for subsequent shape analysis.

**Figure 11:**
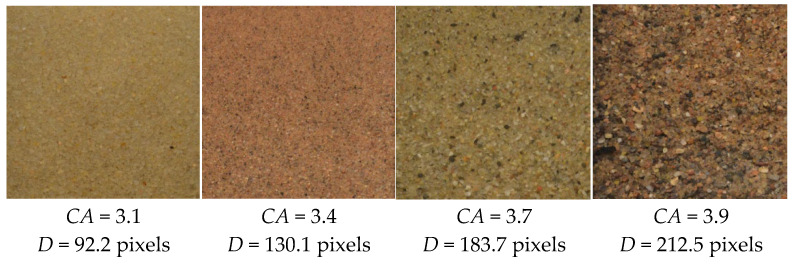

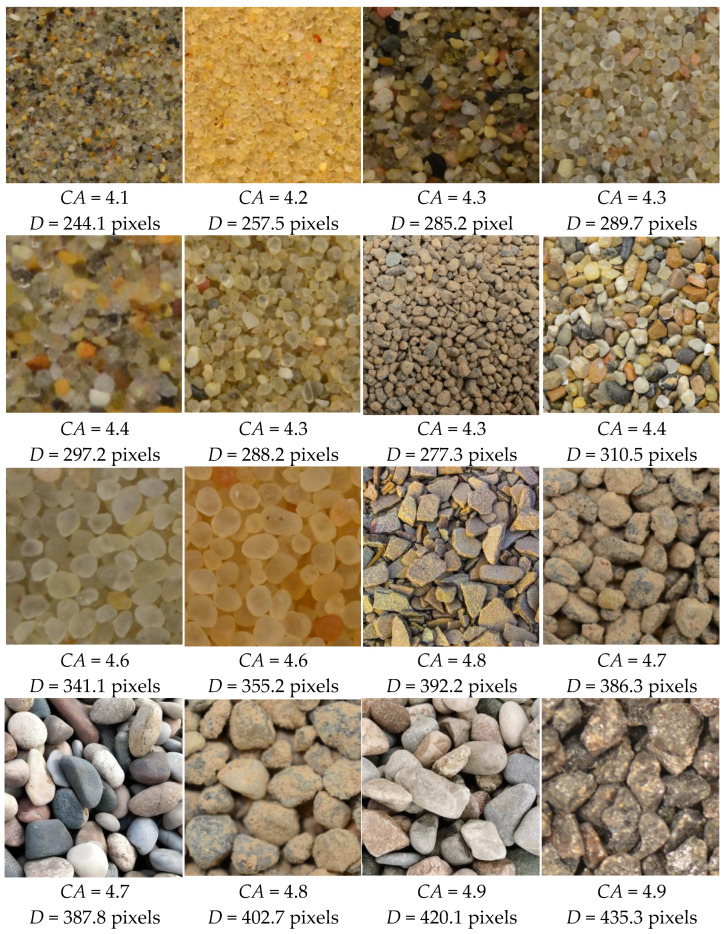
Images of representative soil particles with corresponding *CA* and *D* values.

To further explore the robustness of this approach, wavelet analysis was conducted on 100 high-resolution (4096 × 4096) soil images of varying particle shapes, colors, and sizes. For each image, the composite coefficient *CA* was computed using the Haar transform, while the average particle size *D* was manually measured. As illustrated in Figure 11, the results reinforce the statistical association between *CA* and *D*, demonstrating consistent growth in *CA* values with increasing particle size.

**Figure 12:**
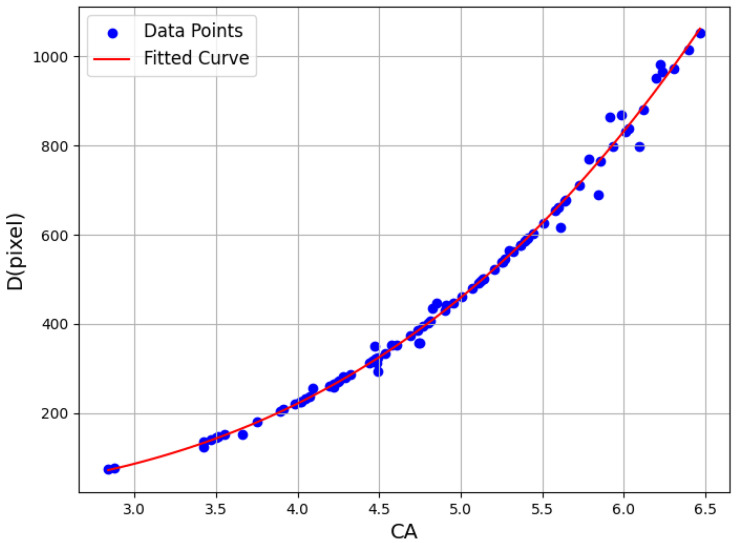
Fitting curve of the relationship between *CA* and *D.*

Figure 12 presents a fitted curve between *CA* and *D*, revealing a nonlinear yet monotonic relationship in which particle size increases at an accelerating rate with respect to *CA*. This correlation confirms that *CA* is a valid and scalable descriptor for particle size, particularly in the context of heterogeneous soil assemblies. It can be expressed as follows:

(12) $$CA={\left(\frac{D}{2.4}\right)}^{\frac{1}{3.3}}$$

As illustrated in the fitted curve (Figure 12), particle size *D* increases at an accelerating rate as *CA* grows, confirming a positive and nonlinear relationship between the two. This supports the use of *CA* as a scale-sensitive indicator for particle size estimation.

Based on this relationship, critical particle sizes, *D*_min_ from Table 1, can be mapped to minimum wavelet thresholds *CA*_min_ required for accurate shape analysis. Table 2 summarizes these values for various shape descriptors. For instance, shape features such as roundness and convexity require a minimum *CA* of 4.1, below which image clarity becomes insufficient for reliable feature extraction.

To automate image quality screening, the process involves resizing input images to 4096 × 4096 pixels, applying a 12-level Haar decomposition to compute *CA*, and comparing the result to *CA*_min_ values according to the target descriptor. This enables resolution-aware filtering of usable images prior to shape extraction.

The effectiveness of this approach was further evaluated using four image reconstruction methods. As summarized in Table 3, Real-ESRGAN outperformed other algorithms, achieving the highest PSNR (24.63 dB), SSIM (0.8402), and *CA* (6.2), indicating superior preservation of morphological fidelity during image enhancement.

In conclusion, the wavelet coefficient *CA* provides a quantitative and objective measure of image quality for particle shape analysis. Its correlation with particle size enables direct conversion of resolution standards into descriptor-specific thresholds, ensuring dependable shape characterization across diverse soil types. This framework supports more efficient and accurate geotechnical modeling, particularly for automated morphological assessment in large-scale image datasets.

### 3.3. Image Quality Evaluation Metrics

To evaluate the performance of the image degradation and reconstruction process, three complementary metrics were employed: Peak Signal-to-Noise Ratio (*PSNR*), Structural Similarity Index Measure (*SSIM*), and Wavelet Coefficient (*CA*). These indicators jointly assess pixel-level fidelity, structural similarity, and multiscale clarity, respectively.

(1) Peak Signal-to-Noise Ratio (*PSNR*)

PSNR quantifies the pixel-wise reconstruction error between a degraded or reconstructed image and the reference image. It is defined as:

(13) $$PSNR=10\cdot \log_{10}\left(\frac{{\left(MAX_{I}\right)}^{2}}{\mathrm{MSE}}\right)$$

where the calculation formula for Mean Squared Error (MSE) is as follows:

(14) $$\mathrm{MSE}=\frac{1}{H\cdot W}\sum_{i=1}^{H}\sum_{j=1}^{W}{\left(I_{\mathrm{gt}}(i,j)-I_{\mathrm{rec}}(i,j)\right)}^{2}$$

Here, $MAX_{I}\;$ is the maximum possible pixel value of the image; $I_{\mathrm{gt}}(i,j)$ is the pixel value of the reference image; $I_{\mathrm{rec}}(i,j)$ is the pixel value of the reconstructed image; and H,W represents the height and width of the image. While *PSNR* is widely used for clarity assessment, it does not always align with perceptual quality.

(2) Structural Similarity Index Measure (*SSIM*)

*SSIM* evaluates luminance, contrast, and structural similarity between two images:

(15) $$SSIM(x,y)=\frac{\left(2\mu_{x}\mu_{y}+C_{1})(2\sigma_{xy}+C_{2}\right)}{\left(\mu_{x}^{2}+\mu_{y}^{2}+C_{1})(\sigma_{x}^{2}+\sigma_{y}^{2}+C_{2}\right)}$$

where $\mu_{x}$, $\mu_{y}$ and $\sigma_{x}^{2}$, $\sigma_{y}^{2}$ represent the means and variances of the images, and $C_{1}$, $C_{2}$ are stabilizing constants. *SSIM* ranges from 0 to 1, with higher values indicating greater structural similarity and perceptual fidelity.

(3) Wavelet Coefficients (*CA*)

Wavelet transform enables multiscale decomposition of an image, capturing spatial variations across different frequency bands and effectively highlighting edge and texture information. As demonstrated by Shin and Hryciw [42], the decomposition depth is closely associated with image clarity. Building on this, the composite wavelet coefficient *CA*, defined in Equation (12), provides a quantitative measure of multiscale structural detail and can be used as an objective indicator of image quality.

### 3.4. Dual-Stage Degradation Results Analysis

To improve the adaptability of the super-resolution reconstruction model, low-resolution training images were generated through a two-step degradation process involving progressive downsampling. This approach ensures that the training dataset captures a wide range of image quality levels and supports learning under both mild and severe degradation scenarios.

**Figure 13:**
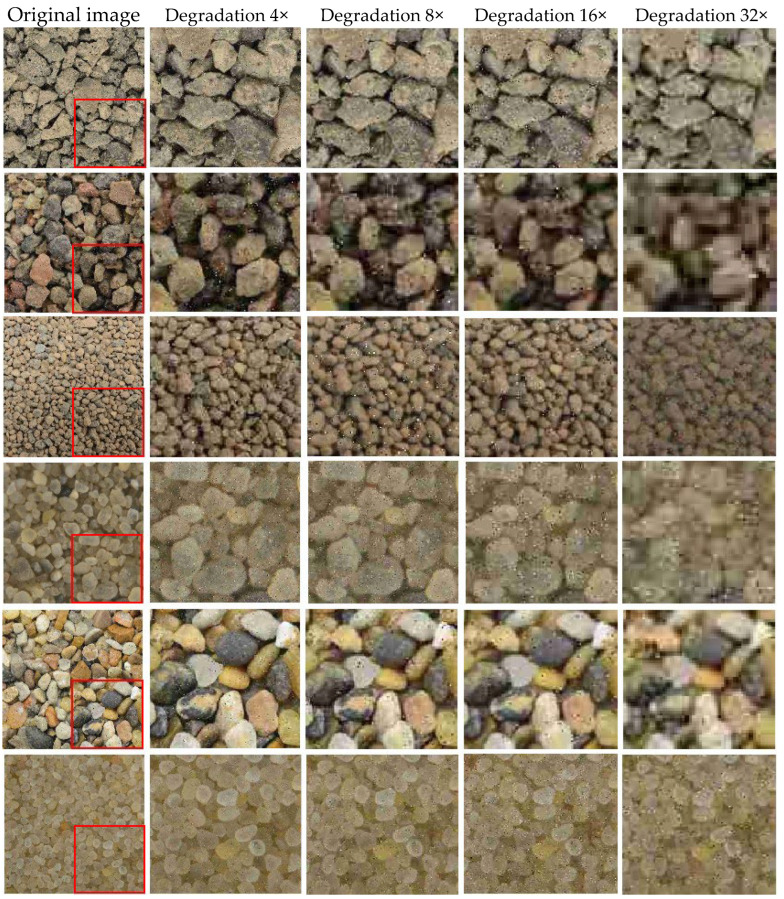
Image Degradation Effects at Different Downsampling Multiples.

As shown in Figure 13, particle images from six roundness categories were degraded at different scales: 4×, 8×, 16×, and 32×. With increasing downsampling factors, the image quality declines significantly. Particle edges become more blurred, noise increases, and fine morphological details are gradually lost. The use of multi-level degradation images supports both effective model training and robustness evaluation under challenging visual conditions.

**Figure 14:**
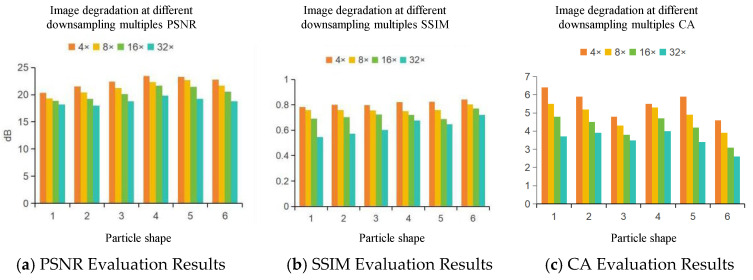
Degraded Image Quality Evaluation Chart.

Figure 14 presents the changes in image quality metrics, including *PSNR*, *SSIM*, and *CA*, across different degradation levels. At 4× downsampling, the images maintain good visual quality, with *PSNR* values above 20 dB, *SSIM* values between 0.75 and 0.8, and *CA* values higher than 4. Most edge features remain clear, and particle shapes can be reliably distinguished. When downsampled at 8×, *PSNR* decreases to 15 to 20 dB, *SSIM* drops to the range of 0.7 to 0.75, and *CA* stays slightly above 4. Although edge blurring begins to appear, roundness features can still be partially recognized.

At 16× degradation, *PSNR* values fall to approximately 17 to 17.5 dB, *SSIM* decreases to between 0.65 and 0.75, and some *CA* values fall below 4. The particle edges become heavily blurred, fine details are lost, and noise becomes more prominent. Distinguishing between different particle shapes becomes increasingly difficult. At 32× degradation, *PSNR* drops below 17 dB, *SSIM* falls under 0.7, and *CA* values are consistently lower than 4. In this condition, image quality is severely reduced, and particle roundness is barely identifiable.

Overall, the results demonstrate that as the downsampling factor increases, all three metrics decline. The CA value shows strong sensitivity to the loss of boundary information, which is important for particle shape analysis. Furthermore, rounded particles tend to maintain higher metric values under the same degradation level. This suggests that smoother contours preserve structural information more effectively than angular ones.

These findings support the selection of training images at 4× and 8× degradation levels, where particle shape features remain recognizable and useful for model learning. Images at 16× and 32× degradation, though of lower quality, can still serve as valuable inputs for testing the model’s performance under more challenging conditions.

## 4. Coarse Granular Particle Image Reconstruction Method for Earth/Rock Dam Construction

In this study, a dual-stage degradation model is used, incorporating multiple iterative degradation processes and utilizing a sinc filter to simulate ringing and overshoot artifacts, thus more comprehensively covering the real degradation space. As all particle images were acquired as single static frames under controlled laboratory conditions, inter-frame super-resolution techniques could not be applied. A single-image super-resolution strategy was therefore adopted to enhance spatial resolution. Real-ESRGAN is a super-resolution image reconstruction method based on Generative Adversarial Networks (GANs). Through adversarial training between the generator network *G* and the discriminator network *D*, the model is gradually optimized to ultimately achieve a balance between perceptual quality and pixel accuracy. Real-ESRGAN Super-Resolution Reconstruction Network is used to reconstruct the clarity and detail features of low-resolution particle images.

### 4.1. Generator Analysis

**Figure 15:**
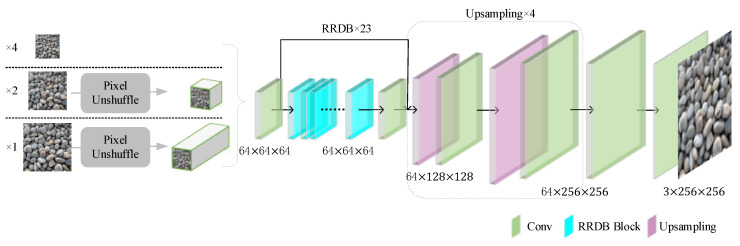
Real-ESRGAN Generator Network Structure Diagram.

The design of generator *G* needs to strike a balance between pixel accuracy and visual perceptual quality. The structure of the Real-ESRGAN generator network is shown in Figure 15. Its objective is to generate a high-resolution image $I_{\mathrm{LR}}$ from a low-resolution image $I_{\mathrm{SR}}$, as described by the following formula:

(16) $$I_{\mathrm{SR}}=G(I_{\mathrm{LR}})$$

The Enhanced Residual Dense Block (RRDB) module combines residual connections and dense connections, enabling efficient extraction and transmission of multiscale features. The formula is as follows:

(17) $$\mathrm{RRDB}(x)=x+\alpha \cdot f_{\mathrm{DenseBlock}}(x)$$

where x represents the input features, $f_{\mathrm{DenseBlock}}(x)$ is the feature extraction function of the dense block, and α is the residual scaling factor used to stabilize the training of deep networks, typically set to 0.1≤α≤1.

In our implementation, the residual scaling factor α was fixed at 0.2 throughout the training process, following the standard configuration in the original Real-ESRGAN setup. This value offers a good trade-off between stable gradient flow and effective feature reuse.

Although Real-ESRGAN was introduced in 2021, it remains one of the most effective frameworks for real-world image super-resolution, especially in scenarios involving unknown and complex degradation patterns. Compared to more recent models such as SeeSR, SeD, and diffusion-based methods, Real-ESRGAN offers a better trade-off between computational efficiency and reconstruction quality, which is critical for engineering applications where real-time analysis and practical deployment are required. Its residual-in-residual dense architecture also ensures robustness in preserving high-frequency particle boundary details. Therefore, this study adopts Real-ESRGAN as a reliable and lightweight baseline, with potential for further enhancement by integrating newer techniques in future work.

### 4.2. Discriminator Analysis

**Figure 16:**
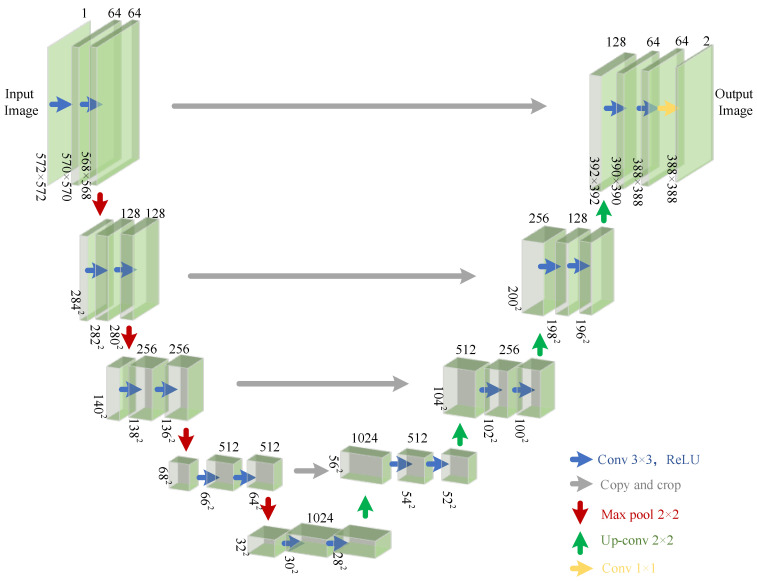
U-Net Discriminator Structure.

To address the problem of real-world complex degradation scenarios, a U-Net discriminator with Spectral Normalization (SN) is used in the adversarial network. The structure’s encoder extracts multiscale features, performing layer-by-layer downsampling while retaining global context information. The decoder uses skip connections to fuse local texture details, providing more precise gradient feedback, enabling the discriminator to distinguish the authenticity of each generated pixel and achieving fine-grained supervision. The specific structure diagram is shown in Figure 16. Real-ESRGAN achieves a balance between local detail enhancement and artifact suppression through the U-Net structure and SN constraints.

### 4.3. Loss Function

(1) Pixel Loss

**Figure 17:**
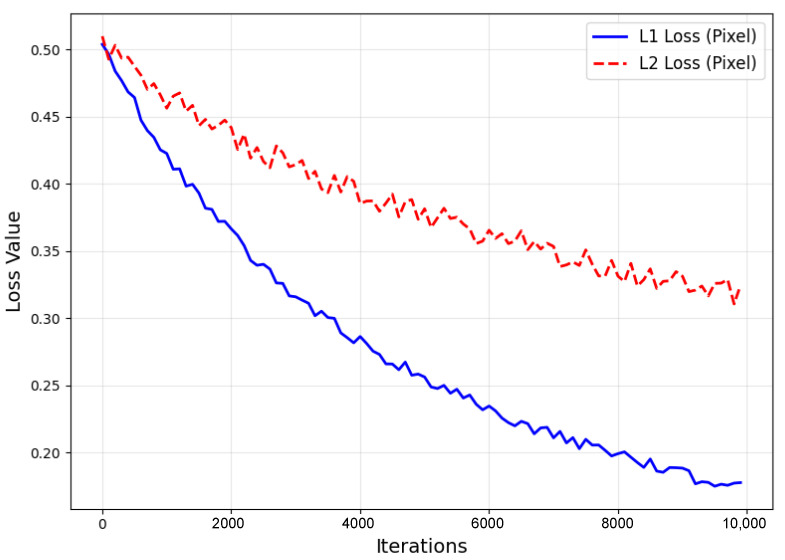
Iteration Curves of L1 Norm and L2 Norm Pixel Loss.

Pixel loss calculates the pixel-level difference between the generated high-resolution image and the true high-resolution image (Figure 17). It is generally represented by either the absolute error loss $L_{pixel1}$ or the squared error loss $L_{pixel2}$, as shown in the following formula:

(18) $$L_{pixel1}(I_{SR},\;I_{HR})=\frac{1}{hwc}\sum_{k=1}^{c}\sum_{i=1}^{h}\sum_{j=1}^{w}|{\left(I_{SR}\right)}_{i,j,k}-{\left(I_{HR}\right)}_{i,j,k}|$$

(19) $$L_{pixel2}(I_{SR},\;I_{HR})=\frac{1}{hwc}\sum_{k=1}^{c}\sum_{i=1}^{h}\sum_{j=1}^{w}{\left({\left(I_{SR}\right)}_{i,j,k}-{\left(I_{HR}\right)}_{i,j,k}\right)}^{2}$$

where h,w,c represent the height, width, and channels of the image, respectively; $I_{SR}$ represents the generated super-resolution image; and $I_{HR}$ represents the true high-resolution image. The advantage of pixel loss is its simplicity and ease of computation. However, its disadvantage is that it neglects high-order image features, which can lead to overly smooth generated images that lack detail and sharpness.

(2) Perceptual Loss

Perceptual loss optimizes the sensory quality of the generated image by comparing the similarity between the generated image and the true image in a high-level feature space.

(20) $$L_{perceptual}(I_{SR},\;I_{HR})=\frac{1}{hwc}\sum_{i,j,k=0}^{h-1,w-1,c-1}\left|\phi {\left(I_{SR}\right)}_{i,j,k}-\phi {\left(I_{HR}\right)}_{i,j,k}\right|$$

where ϕ is the feature extraction network, h,w,c represent the height, width, and channels of the image, respectively; $I_{SR}$ represents the generated super-resolution image; and $I_{HR}$ represents the true high-resolution image.

The advantage of perceptual loss is that it can capture high-order features of the image, improving the visual quality of the generated image. The downside is that it requires an additional feature extraction network and the choice of this network can influence the results.

(3) Adversarial Loss

The objective of the discriminator D is to distinguish between the generated image $I_{SR}$ and the true high-resolution image $I_{HR}$. The loss function is defined as:

(21) $$L_{GAN}=-E_{I_{HR}\sim p_{\mathrm{data}}}[\log D(I_{HR})]-E_{I_{SR}\sim p_{G}}[\log(1-D(I_{SR}))]$$

where $I_{HR}∼p_{data}$ represents the real image sampled from the true distribution $p_{data}$, and $I_{SR}∼p_{G}$ represents the generated image sampled from the generated distribution $p_{G}$. The advantage of adversarial loss is that it can enhance the contrast and sharpness of the generated image, improving its perceptual quality. The downside is that it is difficult to train, and issues such as mode collapse and vanishing gradients can arise.

The total loss function of Real-ESRGAN consists of the following three parts:

(22) $$L_{\mathrm{total}}=L_{\mathrm{pixel}}+\lambda_{1}L_{\mathrm{perceptual}}+\lambda_{2}L_{GAN}$$

where $L_{\mathrm{pixel}}$ is the pixel loss, ensuring pixel-level consistency between the generated image and the true image; $L_{\mathrm{perceptual}}$ is the perceptual loss, optimizing high-level semantic consistency; $L_{\mathrm{GAN}}$ is the adversarial loss, enhancing the visual authenticity of the generated image; and $\left(\lambda_{1},\lambda_{2})\in [0,1\right]$ is the weight parameter.

In our experiments, the loss weights were empirically set to λ_1_ = 0.8 and λ_2_ = 0.2, prioritizing perceptual quality while preserving pixel-wise accuracy. This balance was crucial for achieving both sharp textures and faithful structural restoration.

The total loss function $L_{\mathrm{total}}$ achieves detailed, visually realistic super-resolution image generation in real degradation scenarios by reasonably balancing these three losses.

**Figure 18:**
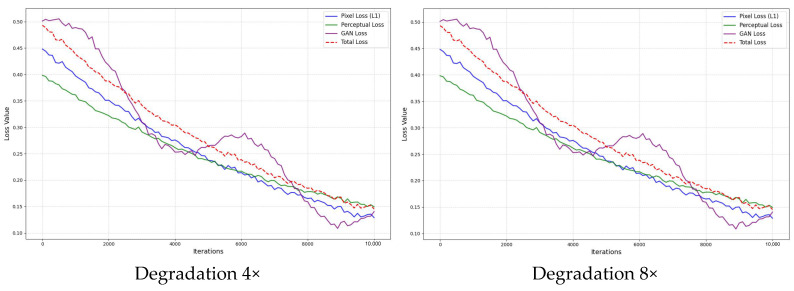

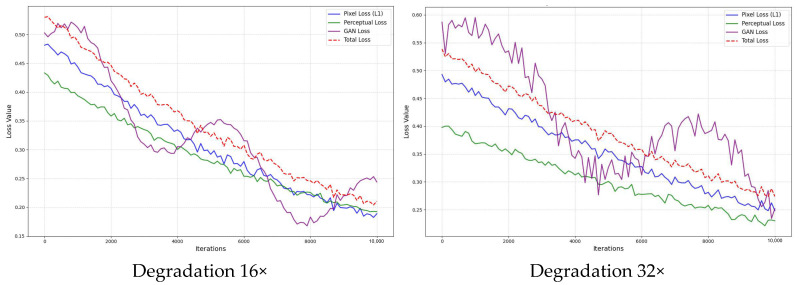
Real-ESRGAN Loss Curves.

As shown in Figure 18, through the analysis of training results at different degradation multiples, the following conclusions can be drawn. In the case of 4× degradation, the model demonstrates the best training performance. Specifically, as the training iterations progress, pixel loss, perceptual loss, and GAN loss all decrease at a relatively steady rate, and the total loss curve shows a significant decline. This indicates that under this degradation condition, the model can efficiently recover clearer details from the degraded images, and the training process is relatively stable with a fast convergence rate.

In comparison, as the degradation multiple increases (such as 8×, 16×, and 32×), the training difficulty of the model gradually increases. At these higher degradation multiples, although the loss curves eventually show a decrease, the rate of decrease is smaller, and there are larger fluctuations during training, resulting in slower convergence and poorer training stability. This indicates that higher degradation multiples present greater challenges for the model in recovering the image, especially in recovering high-frequency details and fine features, with the results not being as effective as those at 4× degradation.

A comprehensive analysis leads to the conclusion that at 4× degradation, the model converges the fastest, exhibits the best stability, and can effectively restore image quality. In contrast, the training process at higher degradation multiples is more complex, and the restoration performance is significantly impacted. Therefore, 4× degradation is the ideal degradation setting in this study, as it achieves high training efficiency and delivers the best performance in image restoration quality.

## 5. Experimental Results and Analysis

### 5.1. Image Reconstruction Results

To validate the effectiveness of the Real-ESRGAN algorithm in particle image reconstruction, this study compares it with mainstream SR algorithms such as SRGAN, SRResNet, and ESRGAN. A test set consisting of 15 images with different particle shapes and roundness was selected, and the reconstruction performance of each algorithm was evaluated both quantitatively and qualitatively. The results are shown in Table 3, where Real-ESRGAN outperforms the other algorithms in terms of PSNR and SSIM metrics, demonstrating its excellent performance in overall image quality recovery.

**Figure 19:**
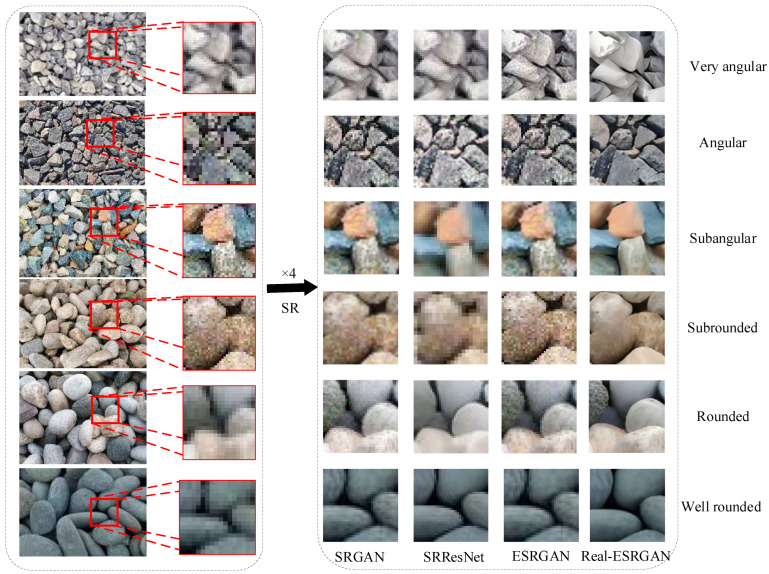
Comparison of SR Reconstruction Results of Different Algorithms on Particle Images with Different Roundness.

As shown in Figure 19, in the reconstruction comparison of particle images with six different roundness categories, Real-ESRGAN exhibited significant advantages over SRGAN, SRResNet, and ESRGAN in edge clarity and detail preservation. Specifically, the images reconstructed by Real-ESRGAN feature sharper edges, stronger noise suppression, and significantly better reconstruction of particle edge textures and shape contours compared to other algorithms. This indicates that Real-ESRGAN, while focusing on high-frequency detail reconstruction, can better balance perceptual quality and noise suppression, leading to more precise restoration of particle image features.

Moreover, in terms of perceptual quality based on subjective evaluation, the images reconstructed by Real-ESRGAN are visually closer to the true images, with particularly impressive restoration of particle details and complex textures. In contrast, SRGAN and ESRGAN exhibit some degree of over-smoothing or texture distortion, while SRResNet tends to generate overly smooth images, resulting in the loss of image details. These results further confirm the superiority of the Real-ESRGAN algorithm in particle image resolution reconstruction tasks.

In conclusion, Real-ESRGAN not only has significant advantages in quantitative metrics such as PSNR and SSIM but also excels in edge clarity and perceptual quality. The superior performance of this algorithm makes it more applicable in particle image resolution reconstruction research, especially in scenarios requiring high-detail restoration and precise feature preservation, where Real-ESRGAN is an ideal choice.

**Figure 20:**
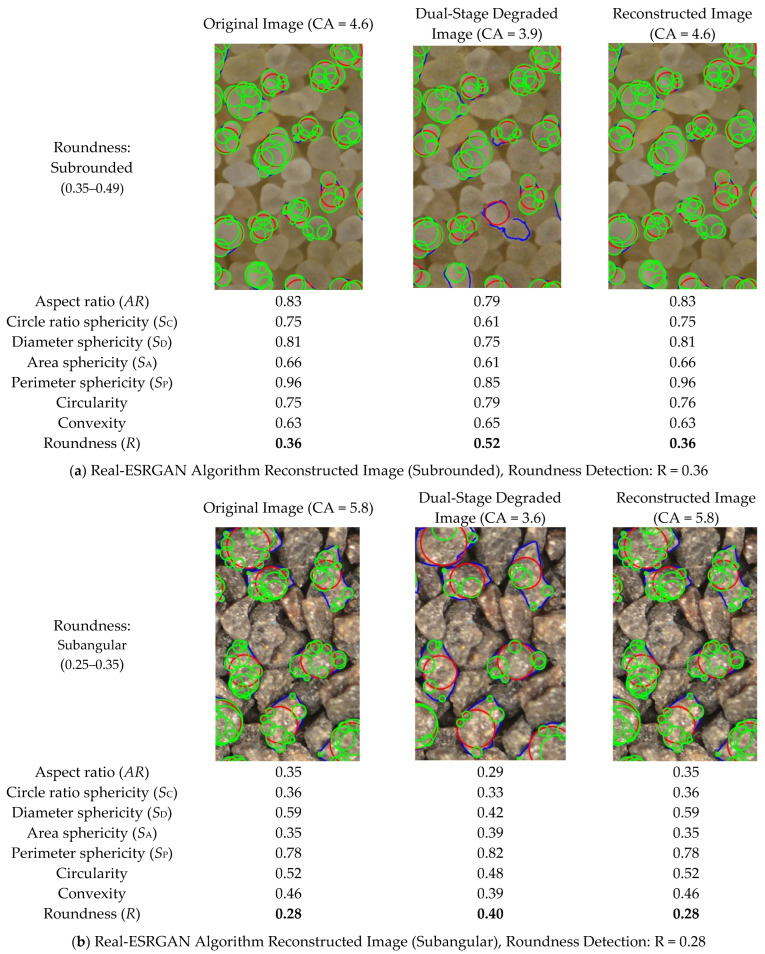

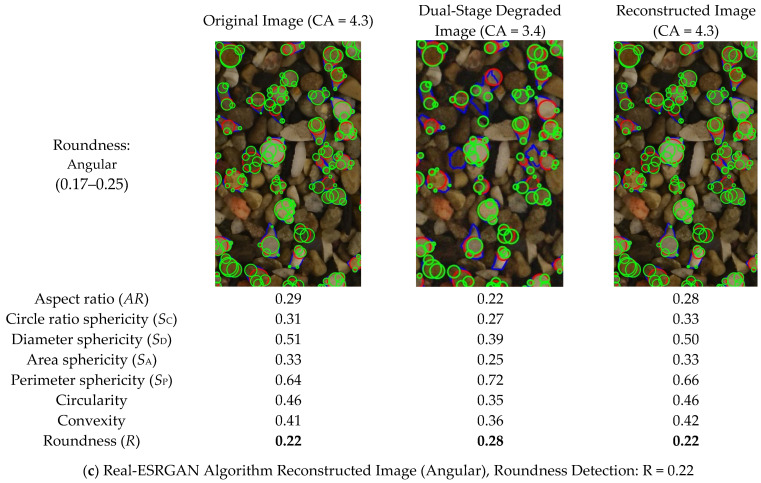
Wadell Roundness Detection Results (Fitted Corner Circle in Green, Maximum Inscribed Circle in Red, Particle Contour in Blue).

Figure 20 shows the process of restoring particle images with different shapes after dual-stage degradation processing using the Real-ESRGAN super-resolution reconstruction algorithm. The particles are classified into three types, each with a different roundness range, as follows:

Subrounded: Roundness between 0.35 and 0.49, with relatively rounded particles and some remaining angularity on the surface.

Subangular: Roundness between 0.25 and 0.35, with irregular particle shapes and sharper angles.

Angular: Roundness between 0.17 and 0.25, with particles exhibiting obvious angles and a more complex shape.

In addition to classical models like SRGAN and ESRGAN, we also reviewed several recent real-world SR methods, such as SeeSR, One-Step Diffusion SR, and SeD. These models demonstrate superior performance on perceptual benchmarks by incorporating semantic priors or iterative denoising mechanisms. However, they typically require pre-trained semantic maps or multiple denoising steps, which increases computational overhead and limits their usability in field-based, real-time applications. In contrast, Real-ESRGAN performs consistently across diverse degradation types while maintaining low latency, making it a more practical choice for coarse particle image enhancement in geotechnical scenarios.

### 5.2. Detailed Analysis

By comparing the morphological feature changes of each particle type in the original, degraded, and reconstructed images, a detailed analysis can be made of the performance of Real-ESRGAN in restoring particle image quality and details.

#### 5.2.1. Original Image

The original image shows the particle images that have not undergone any degradation processing. The shape and details of the particles are clearly visible. The geometric features of each particle type exhibit ideal values at this stage, with high morphological metrics such as aspect ratio, roundness, and sphericity, indicating well-defined particle shapes and rich details.

#### 5.2.2. Dual-Stage Degraded Image

During the image degradation process, the quality of the particle images significantly decreases. The dual-stage degradation model simulates factors such as blurring, noise, and downsampling that may occur during image acquisition and transmission. The particle edges become blurry, details are lost, and the morphological features become unclear. Morphological metrics such as aspect ratio, roundness, and sphericity all decrease, reflecting the degradation of particle shapes. The impact of degradation is particularly noticeable in subangular and angular particles, where the edges and details of the particles nearly vanish.

#### 5.2.3. Reconstructed Image

After super-resolution reconstruction with Real-ESRGAN, the image quality shows significant improvement. The edges of the particles are restored with better clarity, and some details are recovered. Although the restored quality has not fully returned to the level of the original image, the particle shape and details show significant improvement compared to the degraded image. For subrounded particles, the restoration effect is relatively ideal, with roundness and sphericity metrics close to the original image. However, for subangular and angular particles, although the image quality has been partially restored, the restoration effect is still inferior to the original image, particularly in the corners of the particles, where the recovery is weaker.

The morphological metrics in the image detail the changes in particles at different image processing stages. These metrics include the following:

Aspect Ratio (AR): In the original image, the particles have a high aspect ratio, indicating regular shapes. After degradation, the aspect ratio decreases, and the particle shapes become more irregular. After reconstruction, the aspect ratio is partially restored, but it remains lower than the original image, indicating that some shape features are still missing.

Circle Ratio (Sphericity): Sphericity indicates the roundness of the particles. In the original image, the sphericity is relatively high. After degradation, the sphericity value decreases significantly, and the particles’ shapes become irregular. After Real-ESRGAN reconstruction, the sphericity is partially restored, especially in subrounded particles, where the sphericity is close to the original image.

Diameter Sphericity (SB), Area Sphericity (SA), Perimeter Sphericity (SP): These metrics reflect the spherical characteristics of the particles. In the degraded image, all of these morphological metrics decrease significantly, indicating the loss of spherical features and a decline in image quality. After reconstruction, these metrics improve, but the recovery is limited, especially for more complex particle shapes (such as subangular and angular particles), where the restoration effect is poor.

Roundness (R): The roundness value increases in the degraded image, especially in angular particles, where the roundness value approaches subrounded, showing the impact of degradation on particle shape. In the reconstructed image, the roundness improves, but it remains lower than in the original image, indicating that the corner details of the particles have not been fully restored.

It is clear that Real-ESRGAN demonstrates significant superiority in restoring different types of particle images, especially in the recovery of subrounded particles, where particle details and geometric features are well restored.

## 6. Conclusions

The experimental results clearly demonstrate that the Real-ESRGAN super-resolution reconstruction method offers significant advantages in enhancing the quality of low-resolution images. The experiments show that after processing with Real-ESRGAN, the reconstructed images are not only visually clearer and more detailed but also more closely match the actual geometric features of the particles in objective evaluation metrics, such as Wadell roundness detection.

In low-resolution images, due to the impact of degradation conditions, the geometric information of the particles cannot be accurately represented, leading to deviations between the roundness detection results and the actual characteristics. However, Real-ESRGAN, through the dual-stage degradation model and detail optimization strategy, effectively restores the high-frequency features of the image, allowing the reconstructed image to more realistically reflect the boundaries and texture details of the particles, thereby improving the accuracy of roundness detection. This finding further validates the practical application value of high-resolution reconstruction technology in image analysis.

Furthermore, comparisons across different experimental groups show the stability of image reconstruction in complex scenarios, especially when particle boundaries are blurry or noise is present. Even under such conditions, Real-ESRGAN can still accurately reproduce the boundary shape of the particles, providing a reliable foundation for subsequent feature extraction and classification.

In summary, Real-ESRGAN technology provides an efficient and practical solution for the precise analysis of complex particle images, highlighting its broad prospects in the engineering applications of earth/rock mixtures.

## Figures and Tables

**Table 1:** The minimum requirements for ensuring accurate particle shape characterization (measured in pixels).

Shape Descriptors		*D*_min_	Hierarchy
Aspect ratio (*AR*)		25	Coarse descriptors: Assessing the main dimensions of particles
Circle ratio sphericity (*S*_C_)			
Diameter sphericity (*S*_D_)		100	Medium-coarse descriptor: Pertaining to the areas of particles
Area sphericity (*S*_A_)			
Perimeter sphericity (*S*_P_)		130	Fine descriptor: Pertaining to the perimeter of particles
Circularity			
Convexity		250	Very fine descriptors: Assessing the perimeters of particles
Roundness (*R*)	Very angular to angular (0 < *R* < 0.17)		
	Angular to rounded (0.17 < *R* < 0.70)	130	
	Rounded to well-rounded (0.70 < R < 1.0)	75	

**Table 2:** Minimum wavelet coefficient values for accurate characterization of particle shape.

Shape Descriptors		*CA*_min_	Hierarchy
Aspect ratio (*AR*)		2.1	Coarse descriptors: Assessing the main dimensions of particles
Circle ratio sphericity (*S*_C_)			
Diameter sphericity (*S*_D_)		3.1	Medium-coarse descriptor: Pertaining to the areas of particles
Area sphericity (*S*_A_)			
Perimeter sphericity (*S*_P_)		3.4	Fine descriptor: Pertaining to the perimeter of particles
Circularity			
Convexity		4.1	Very fine descriptors: Assessing the perimeters of particles
Roundness (*R*)	Very angular to angular (0 < *R* < 0.17)		
	Angular to rounded (0.17 < *R* < 0.70)	3.4	
	Rounded to well-rounded (0.70 < R < 1.0)	2.9	

**Table 3:** Image Reconstruction Results Evaluation.

Reconstruction Method	Evaluation Metrics		
	PSNR/dB	SSIM	CA
SRGAN	23.03	0.7606	4.4
SRResNet	23.34	0.7764	4.9
ESRGAN	23.37	0.7740	5.4
Real-ESRGAN	24.63	0.8402	6.2

## Data Availability

The raw data supporting the conclusions of this article will be made available by the authors on request.
